# Can Machines Learn Respiratory Virus Epidemiology?: A Comparative Study of Likelihood-Free Methods for the Estimation of Epidemiological Dynamics

**DOI:** 10.3389/fmicb.2018.00343

**Published:** 2018-03-02

**Authors:** Heidi L. Tessmer, Kimihito Ito, Ryosuke Omori

**Affiliations:** ^1^Division of Bioinformatics, Research Center for Zoonosis Control, Hokkaido University, Sapporo, Japan; ^2^Precursory Research for Embryonic Science and Technology (PRESTO), Japan Science and Technology Agency, Saitama, Japan

**Keywords:** respiratory virus, infectious disease epidemiology, machine learning, approximate Bayesian computation, basic reproduction number, mathematical model

## Abstract

To estimate and predict the transmission dynamics of respiratory viruses, the estimation of the basic reproduction number, *R*_0_, is essential. Recently, approximate Bayesian computation methods have been used as likelihood free methods to estimate epidemiological model parameters, particularly *R*_0_. In this paper, we explore various machine learning approaches, the multi-layer perceptron, convolutional neural network, and long-short term memory, to learn and estimate the parameters. Further, we compare the accuracy of the estimates and time requirements for machine learning and the approximate Bayesian computation methods on both simulated and real-world epidemiological data from outbreaks of influenza A(H1N1)pdm09, mumps, and measles. We find that the machine learning approaches can be verified and tested faster than the approximate Bayesian computation method, but that the approximate Bayesian computation method is more robust across different datasets.

## Introduction

Prediction of infectious disease epidemics is essential to their control, but also a difficult process. This is because the epidemiological dynamics, i.e., the time evolution of the number of infected individuals, are non-linear, with the probability of a susceptible individual acquiring infection depending on the number of infected individuals. Previous studies have constructed mathematical models describing the transmission dynamics of infectious disease, known as the Susceptible-Infectious-Removed (SIR) model, and fit the model to the time series data of the number of infected individuals (Bjrnstad et al., 2002). Conventional statistical methods, e.g., maximum likelihood estimation, require explicit solution of the time series data of the number of infected individuals from the SIR model. However, an explicit solution is difficult to obtain due to the nonlinearity of the model. Therefore several approximations are required to fit the SIR model with the epidemiological data of infectious diseases. Furthermore, the transmission of infectious disease is a stochastic event. A mathematical model taking into account stochasticity is required to estimate parameters.

One common property of transmission dynamics is the threshold for outbreak: an outbreak occurs only if the basic reproduction number, *R*_0_, exceeds unity. In a biological sense, *R*_0_ is the expected number of secondary infections by an infected individual when a population is fully susceptible (Diekmann et al., 1990). Estimation of *R*_0_ helps to predict the outbreak potential, final epidemic size, timing of the epidemic peak, and vaccination coverage required to prevent an outbreak. To estimate *R*_0_, a common method is to fit the SIR model to epidemiological data. The simplest SIR model has only this one parameter, *R*_0_, by scaling the unit time in the SIR model. In this paper, we use a Susceptible Exposed Infectious Removed (SEIR) model, a variation of the SIR model. The SEIR model is comprised of additional parameters and follows more complex epidemiological dynamics, which reflect realistic disease dynamics.

Due to the importance of estimating *R*_0_, numerous methods have been developed (Magal and Ruan, 2014). The accuracy of the estimates depends on both the estimation method and the data. For example, in one approach *R*_0_ can be estimated from the slope of the time series data of infected individuals at the initial phase of an epidemic (Nishiura et al., 2009). This method approximates the epidemiological dynamics at the initial phase as an exponential growth. The accuracy of this method is sensitive to the period of epidemiological data available. An alternative approach estimates *R*_0_ from the final epidemic size, i.e., the total number of infected individuals (Vynnycky et al., 2007). Because the relationship between the final epidemic size and *R*_0_ cannot be described explicitly, the likelihood function of *R*_0_ with an arbitrary final epidemic size cannot be described in an explicit form. Consequently numerical solutions or approximations are required to construct the likelihood function.

Recently a likelihood-free method has been proposed: approximate Bayesian computation (ABC) (Sunnåker et al., 2013; Saulnier et al., 2017). This method approximates the posterior distribution using a rejection algorithm with the numerical integration of the SEIR model. This method is easy to implement, however several limitations remain. Some issues include (a) parameter estimation takes a long time, particularly as the epidemiological model complexity increases and (b) the ABC method accuracy is dependent on both the summary statistic and the accept/reject decision threshold, but there are no fixed rules for the selection of either.

A second likelihood-free approach has recently emerged in the form of machine learning (ML). The field of machine learning has grown rapidly with a large expansion of theories, applications, and algorithms. Problems can be categorized as either supervised or unsupervised and as classification or regression (Bishop, 2006). A supervised learning problem has a dataset and an answer, for example the pixels making up a photograph of a number can be a dataset and the numerical representation of the number is the answer (e.g., the number “7”). These two pieces of information are passed to the ML model during training so the model learns to recognize pixels of the type given as the answers it receives. Once the model is trained, a separate, new dataset is given which contains only the pixel information. The model is then asked to predict the answer based on the data it had previously seen. This example of supervised learning is also an example of a classification problem. The number problem can be split into 10 discrete categories (the whole numbers “0”–“9”), and the machine learns to classify the results into these categories. A regression problem, on the other hand, seeks to find a continuous value answer to the input it receives. Predicting housing prices is a common example of a regression problem, where, given a set of information about properties, the ML model can predict a continuous, numerical value estimate for the cost of the property.

Supervised ML models combine linear regression, gradient descent, maximum likelihood, and least squares functions to develop weight matrices and comparison functions to predict and estimate parameter outputs based on the historical knowledge of input/output pairs (Bishop, 2006). With the expansion of the field of ML, new models continue to be developed and improved, connecting the building blocks of ML in new ways to uncover hidden connections in data. Some methods, such as convolutional neural networks (CNN) are well suited for two-dimensional image analysis (Krizhevsky et al., 2012), while other methods, like long-short term memory models (LSTM), specialize in handling time series data (Hochreiter and Schmidhuber, 1997).

In this study, we propose a ML approach to estimate the *R*_0_ of a respiratory virus from a time series of incidences of the disease as a supervised regression problem. Additionally, we seek to estimate other parameters associated with the SEIR model and time series generation. As mentioned above, *R*_0_ is highly dependent on the mathematical model. Our final goal is a likelihood-free estimation of *R*_0_, as well as other model parameters. The ML methods used in this study are two separate multi-layer perceptrons (MLP), a CNN, and a LSTM model. For reference and comparison, we also use the ABC approach to estimate the same values using the same datasets. We compare not only the accuracy of the two methods, with credible and confidence intervals, but also the time required by each approach to reach its answer. Of the four ML methods tested, the MLP with time model was the most robust as well as being significantly faster than the more complex CNN and LSTM models.

## Materials and methods

This study can be broken into five main parts. The first is the development of an individual-based (IBM) SEIR epidemiological model for generating data; the second is the ABC method used for estimating parameters; the third is the learning by MLP, CNN, and LSTM machine learning models, again to estimate parameters; the fourth is the dataset creation and bootstrapping of the real-world and test data to create confidence intervals on the machine learning solutions; and finally calculation of the time it took for each method to obtain its estimates.

The ML models were trained on 100,000 datasets, validated on 1,000 datasets, and tested on 1,000 datasets. ABC was run on 1,000 sample datasets and compared against a total of 100,000 comparison datasets. The ABC sample and ML test datasets were the same and the ABC comparison and ML training datasets were the same. An explanation of these datasets is in the following section. We evaluate the accuracy of estimation by two measurements, the average error and the width of the credible interval for ABC and confidence intervals for ML. Each parameter range was divided into 10 subranges. The errors among parameters in each subrange were then averaged to create the “average error.” The average width of credible/confidence intervals is the average difference between the lower and upper bound of the interval.

### Terminology

As the different likelihood-free methods used in this paper (ABC and ML) each have their own standard vocabularies, we first clarify terminology in this paper to make a direct comparison of methods possible.

ML typically uses three datasets. The “training” set is a large dataset which is given to the ML model during the learning phase. The “test” set is a completely new and unseen dataset which the ML method passes through the trained model to estimate the posterior parameter values. The “validation” or “development” dataset, like the “test” set is a new and unseen dataset used as an interim test. That is, this dataset is used to verify the model is learning, check accuracy of estimates, and when running trials of different hyperparameter sets. In ABC there is no dataset equivalent to ML's validation set.

According to the notation used by Sunnåker et al. (2013) we use the symbols *D* for the data in question, either real-world observed data or generated “test” data, and $\hat{D}$ for comparison data. In ABC, $\hat{D}$ would normally be the Markov chain Monte Carlo generated data presented to the rejection algorithm. The rejection algorithm takes a set of generated data and compares it to the data in question, *D*. In ABC, the summary statistic is used to calculate the distance between $\hat{D}$ and *D*, and the parameters are accepted or rejected if the estimated distance falls beneath an accept/reject threshold. Our summary statistic for ABC is the Euclidean distance between the dataset *D* and the simulated dataset $\hat{D}$:

$$\sqrt{\sum {\left(D-\hat{D}\right)}^{2}}$$

Comparing the ML and ABC terminology, datasets comprising *D* would be the test set in ML, while a dataset comprising $\hat{D}$ is given to an ML algorithm as a training set. To compare the ABC and ML methods we use the same pre-generated datasets for both methods.

### SEIR epidemiological model

The SEIR model is an expansion of the SIR model, which describes the time evolution of the number of infected individuals during a disease outbreak. The host population is classified by their health status, susceptible (*S*), exposed (*E*), infectious (*I*), and removed (*R*) (recovered or deceased). Transmission events happen via contact of *S* and *E* with constant rate β. The SEIR model can be expressed mathematically through the following simple equations (Bjrnstad et al., 2002; Diekmann et al., 2009; Magal and Ruan, 2014):

$$\begin{array}{l} N=S(t)+E(t)+I(t)+R(t),\\ \frac{dS}{dt}=-\beta S\frac{I}{N},\\ \frac{dE}{dt}=\beta S\frac{I}{N}-\epsilon E,\\ \frac{dI}{dt}=\epsilon E-\gamma I,\\ \frac{dR}{dt}=\gamma I. \end{array}$$

Here *N* describes the total host population size. In this model, *R*_0_ is given by:

$$R_{0}=\frac{\beta }{\gamma }.$$

This is a deterministic model and *S*, *E*, *I*, and *R* are continuous. To fit the model with the data, it is required to expand this model to a stochastic one with discrete *S*, *E*, *I*, and *R*. An individual-based SEIR model describes the stochastic process of transmission dynamics at the individual level. Let *H*_*x*_ be the health state of the *x*-th individual.

$$H_{x}\in \{S,E,I,R\}$$

The probability of transition between each health state can be written by:

$$\begin{array}{l} Pr(H_{x}(t)=S\to H_{x}(t+\Delta t)=E)=\beta I(t)\Delta t,\\ Pr(H_{x}(t)=E\to H_{x}(t+\Delta t)=I)=\epsilon \Delta t,\\ Pr(H_{x}(t)=I\to H_{x}(t+\Delta t)=R)=\gamma \Delta t. \end{array}$$

We simulate this model to create data which can be used to learn the different parameter sets of *R*_0_, ϵ, and γ. ϵ is the latent period, or the rate at which exposed individuals become infectious. γ is the recovery rate, the rate individuals move from state *I* to state *R*. The host population size for the general study, *N* = 2225. For the real-world comparisons, *N* = 500 for mumps (Sullivan et al., 1985), *N* = 343 for measles (Mossong and Muller, 2000), and *N* = 2,225 for influenza A(H1N1)pdm09 (Lessler et al., 2009). We set *S*(0) = *N* − *I*(0), *E*(0) = 0, *I*(0) = 22, and *R*(0) = 0 as the initial conditions, which were parameterized based on the epidemiological data of Lessler et al. (2009). Time series data of incidence were created by IBM model with randomly chosen parameter sets to consist of 100,000 training, 1,000 validation, and 1,000 test samples. Throughout this study we set Δ*t* = 1/10 day, meaning the SEIR model parameters were updated 10 times each day, with each change in time (Δ*t*) equal to 1/10 day.

### Approximate Bayesian computation

**Figure 1:**
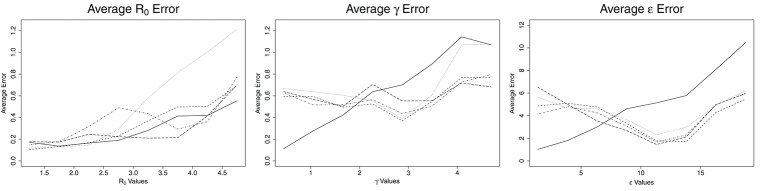
Average error for ABC (solid line), CNN (long dashed line), MLP (dotted line), MLP with time (dashed line), and LSTM (dashed and dotted) estimates against actual parameter values for an SEIR model with three parameters, on 100,000 training datasets.

We use ABC to estimate the parameters *R*_0_, ϵ, and γ, the parameter values associated with the SEIR epidemiological model. For our ABC calculations, we used simulated datasets for $\hat{D}$, calculating the distances between each *D* dataset and $\hat{D}$ datasets. The Euclidean distance was used as the summary statistic to calculate distances between datasets, and multiple acceptance thresholds were established. An acceptance threshold of 60 was used for the Figures 1, **3**, **4**, here as it was large enough to produce accepted posterior parameter sets for each of the *D* datasets and have enough samples to create credible intervals, but small enough to generally discriminate between similar and dissimilar datasets.

The Euclidean distance between the dataset *D* and simulated dataset $\hat{D}$ is:

$$\sqrt{\sum {\left(D-\hat{D}\right)}^{2}}$$

### Machine learning

Three separate machine learning models and one variation were implemented and run with early-stopping manually executed by comparing the loss at each epoch, stopping when the loss no longer decreased (for 10 or 20 epochs), and using the weights from the best epoch, i.e., the epoch with the smallest loss. The learning rate for all models was 0.0001. The ML models in this study were implemented in Python using Lasagne and Theano (Bergstra et al., 2010) libraries. For all ML models, numerous hyperparameters for the number of hidden layers, number of hidden units, learning rate, activation functions, and number of training samples were explored and the hyperparameters which routinely yielded the best results were selected. For example, we ran an MLP model with 2, 3, 4, 5, 6, and 7 hidden layers and found that there was little benefit in models deeper than three hidden layers, yet two hidden layers learned poorly. For this reason, three hidden layers were used in the final model.

#### Multi-layer perceptron

The first model selected for this paper was a simple MLP where the entire time series dataset was passed in as a single input array and the parameters were estimated from learned relationships in that dataset. Small changes in parameter values have a large impact on the shape and behavior of the time series graph, so it was important for the MLP to see all of the data with equal importance, which is why the time series was passed in as a single input.

The MLP model accepted the time series information for the number of incidences of infection per day as created by the IBM model as input for learning. This input was given to the model as a single entity consisting of the number of newly infected individuals at each timestep. It also received the “answers” of the *R*_0_, γ, and ϵ model parameters which were used to generate the time series data. The model was asked to learn these answer parameters in a supervised manner given the time series of incidences. The MLP model was created with three hidden layers having 400 hidden units per layer. The hidden layers used rectified linear units (Maas et al., 2013) for their activation functions, with a linear activation function in the final output layer.

#### Multi-layer perceptron with time

A second MLP model was constructed to incorporate the concept of “time” into the time series information. In a standard MLP, the sequence of values passed in has no connection and there is no concept of order in the analysis and training. This second model added a time element by creating a tuple consisting of the day and the number of new incidences that day. This simple method created a model which understands time as an individual value, though unlike LSTM described later, it does not have any kind of memory mechanism to compare previous and future datapoints.

This MLP also had three hidden layers, though with 400, 200, and 100 hidden units per layer, in that order. Again, the hidden layers used rectified linear units for their activation functions with a linear activation function in the final output layer. The addition of the time element changed the input to the model from a one-dimensional array to a two-dimensional matrix.

#### Convolutional neural network

While the CNN is not a traditional choice for problems involving time series data, it was selected in this case due to the complex nature of the SEIR modeling data. The mathematical models being simulated are highly non-linear, nearly chaotic at times. As CNNs are known to be capable of modeling very complex behavior (Krizhevsky et al., 2012), they were tested in this problem space for comparison.

The CNN model was constructed of two one-dimensional convolutional layers and two pooling layers, with a single dense hidden layer prior to the output. The convolutional and hidden layers all used rectified linear units for their activation functions, while the output layer again used a linear activation function.

#### Long-short term memory

A recurrent neural network (RNN) approach, LSTM models are designed for analysis of time series data (Hochreiter and Schmidhuber, 1997), like that found in this study. The memory aspect is built into the model using a memory cell and gates (input, output, and forget) to control the data in the model. These components ensure continuity in the data of the LSTM model and accept the time order as an important feature of the data.

The LSTM model implemented for this study consists of two LSTM layers with 16 hidden units and gradient clipping at 100 (to prevent exploding gradients). No activation function is applied in the LSTM layers, however a linear activation function is applied on the output layer.

### Datasets

The datasets for this study come from two sources. The first, generated data, is a set of SEIR epidemiological model datasets generated using individual-based, Monte Carlo simulations, as described in the Epidemiological Model section. The second source comes from time series sets of incidences from published papers by Lessler et al. (2009), Sullivan et al. (1985), and Mossong and Muller (2000). We estimate *R*_0_ for these time series datasets and compare our estimates against the general or estimated value from the papers.

The data in these datasets are comprised of two parts. The first part is a time series of the number of newly infected individuals per day over the course of an outbreak. The second piece of information is the parameter set of *R*_0_, γ, and ϵ used in the simulation to generate the time series. The time series is the information the ML model trains on, while the parameter set is the answer it is trying to achieve. In ABC, the time series is the dataset $\hat{D}$, and the parameter set is the answer it is estimating.

#### Bootstrap resampling

To date, machine learning has most often been used in classification problems with discrete correct answers and myriad ways to determine the performance of any given model, such as accuracy, precision/recall, F-score, and receiver operator characteristic (ROC) (Sokolova et al., 2006). Regression problems, by contrast, have few methods to explore the quality of the output. To address this issue, we created a novel method to build confidence intervals for the outputs of the ML model.

We started by creating a standard machine learning test set of 1,000 datasets. Running this time series dataset through the trained model gives estimates for the parameter values, but there is no indication on the quality of the estimates—no certainty or confidence associated with the values. Next, we created 1,000 bootstrap-resampled datasets for each test dataset, for a total of one million tests. For bootstrap resampling of the time series data of incidence, the time series data of incidence can be interpreted as the set of emergence times. For example, the data when incidence at *t* = 1 is 1 and incidence at *t* = 2 is 2 is equivalent to a set of emergence times, {*t* = 1, *t* = 2, *t* = 2}. We resampled the emergence times by bootstrap resampling from this set of times, and converted them back into time series data of incidence.

For the estimates returned for the 1,000 resamples of each dataset, we calculated the mean, median, mode, and 95% confidence intervals. This method provides a measure of credibility for each estimated output parameter.

### Time calculations

The ABC computations were conducted on a server with 2.80 GHz processors and 1 TB of memory. The computations were run across multiple CPUs and the time calculated is the combined time to run all scripts. The ML computations were conducted on a server with 3.50 GHz processors, 64 GB memory, and a GeForce GTX 1080 Ti® graphics card for GPU processing. Typically between four and eight processes were run simultaneously on the graphics card.

The time to complete each method is shown in Table 1. For ABC, the time to verify its results by comparison of the distances between 1,000 *D* datasets with 100,000 $\hat{D}$ datasets was measured, including time to split by thresholds and create credible intervals for accepted datasets. The time for a single comparison against 100,000 $\hat{D}$ datasets was then measured for the test comparison. For the ML models, the time included the bootstrapping of the test dataset, training of the learning model, and computation of the confidence intervals. The test was then run by obtaining the estimates for a single bootstrapped sample, that is, running 1,000 samples from one set of parameters through a trained ML model and collating the results.

**Table 1:** The time required to train 100,000 samples and test on a single sample for the ABC, MLP, CNN, and LSTM methods.

**Method**	**Train (min)**	**Test (min)**
ABC	410	0.65
MLP	68	0.03
MLP with time	71	0.03
CNN	184	0.05
LSTM	364	0.06

### Experiments on real-world datasets

For experiments, we used an SEIR model dataset with three parameters: *R*_0_, γ, ϵ, calculating distances with ABC and comparing at various acceptance thresholds, and training CNN, LSTM, and two MLP models, then running a test dataset through the trained model. Finally, we tested our trained and verified models against three real-world datasets: (1) an outbreak of mumps (Sullivan et al., 1985), (2) an outbreak of measles (Mossong and Muller, 2000), and (3) an outbreak of influenza A(H1N1)pdm09 (Lessler et al., 2009) to see if we could accurately estimate the parameter *R*_0_ with both our ABC and ML models.

For ABC, the time series of infectives is set as *D* and compared against the $\hat{D}$ generated data used throughout this paper. The required accept/reject threshold was 300, 20, and 20 for the influenza, measles, and mumps datasets, respectively. The accept/reject thresholds were selected where there were enough accepted datasets for the calculation of credible intervals. The parameters were then estimated from the accepted datasets. To construct confidence intervals of the estimates by ML, the real-world time series of incidence was resampled using the bootstrap resampling method discussed in the Bootstrap Resampling section. The set containing the original and bootstrap resampled time series are given to the model, the parameters are estimated, and finally the confidence intervals are calculated from the ML model outputs.

## Results

### Comparisons of average errors

Figure 1 shows the average errors compared to the actual parameter values associated with each method. The average errors of estimates made with ABC and ML are most similar for *R*_0_. ABC and MLP with time show nearly identical patterns, consisting of low errors when *R*_0_ is <3.0 and then increasing with increasing values of *R*_0_. ABC has slightly lower average error than MLP with time for all values of *R*_0_. For *R*_0_ below 2.25, MLP performs as well as ABC and MLP with time. For *R*_0_ >2.25 the average error from MLP estimates increases greatly, until *R*_0_ reaches 5.0, at which point the error from MLP is approximately twice the error from ABC. CNN has a nearly constant average error of 0.2 for *R*_0_ < 3.8. For *R*_0_ > 3.8, the average error on CNN estimates increases linearly to approximately 0.7 at *R*_0_ = 5.0. Finally, LSTM shows erratic behavior in *R*_0_ estimation. Until *R*_0_ reaches 3.0, LSTM has the worst estimates among the methods tested, with an error reaching nearly 0.6. From 2.8 to 3.8, the average error by LSTM decreases, becoming smaller than all but CNN. From *R*_0_ = 3.8 to 5.0, the error by LSTM increases again, with behavior similar to ABC, CNN, and MLP with time for this range of *R*_0_.

**Figure 2:**
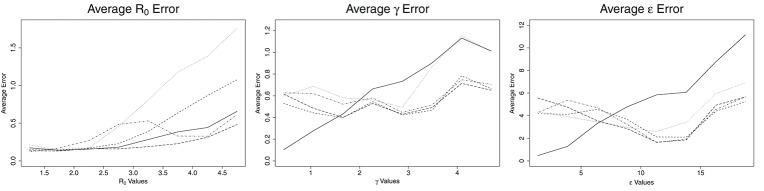
Average error for ABC (solid line), CNN (long dashed line), MLP (dotted line), MLP with time (dashed line), and LSTM (dashed and dotted) estimates against actual parameter values for an SEIR model with three parameters, for one million training datasets.

The patterns for average error for the parameters γ and ϵ are similar to one another. ABC follows one pattern while the ML solutions follow a different pattern. For ABC, the average error increases with increasing values of the parameters, γ and ϵ. For γ < 2.0 days^−1^ and ϵ less than about 7.5 days^−1^, the ABC average error is smaller than the average error for all ML estimates. For γ more than 2.0 days^−1^ and ϵ more than 7.5 days^−1^, the average error for ABC is larger than the average error of all ML estimates. When the number of training samples is increased to one million, the point at which ML becomes more accurate than ABC is 1.6 days^−1^ for γ and approximately 6.5 days^−1^ for ϵ (see Figure 2). The ML approaches maintained a nearly consistent average error for all γ of 0.6 days^−1^ until γ = 3.9 days^−1^, when they increased to average errors of approximately 0.6 days^−1^. From γ = 3.9 to 5.0 days^−1^, MLP increased much more quickly to approximately 1.1 days^−1^, the same as ABC. All four ML models behaved the same for ϵ, with average errors decreasing with increasing ϵ until ϵ = 11 days^−1^, at which point they increased with increasing ϵ at a rate similar to ABC, but approximately 3 days^−1^ smaller.

### Comparisons of credible/confidence intervals

**Figure 3:**
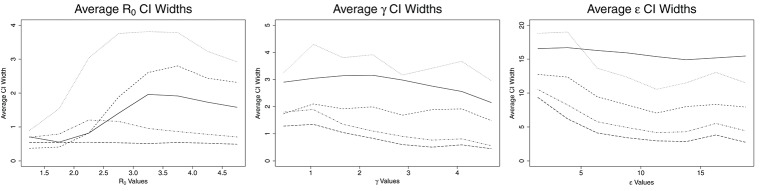
Average credible/confidence interval width for ABC (solid line), CNN (long dashed line), MLP (dotted line), MLP with time (dashed line), and LSTM (dashed and dotted) estimates against actual parameter values for an SEIR model with three parameters, on 100,000 training datasets.

The credible intervals for the estimates for ABC and confidence intervals for ML are shown in Figure 3. CNN has a constant size confidence interval for *R*_0_. MLP and MLP with time have a confidence interval similar in shape to ABC's credible intervals for *R*_0_, though their confidence intervals are larger. These three methods start with small credible/confidence intervals for small *R*_0_, increasing with *R*_0_ until *R*_0_ = 2.6–4.0 and then decreasing slightly. LSTM's confidence interval increases until *R*_0_ reaches approximately 2.25, then decreases with increasing *R*_0_. The credible/confidence intervals for both the ABC and ML models decrease as γ increases. CNN has the smallest confidence interval for γ, followed by LSTM, MLP with time, ABC, and MLP. In ABC, the credible intervals for ϵ remain mostly constant at about 16.0 *day*^−1^ for all values of ϵ. For the ML models, the confidence intervals decrease with increasing ϵ, increasing slightly around ϵ = 15.0 days^−1^ and then decreasing again. Again, CNN has the smallest confidence interval for ϵ, followed by LSTM, MLP with time, MLP (for ϵ > 5 days^−1^), and ABC.

### Comparisons of estimated and actual values

**Figure 4:**
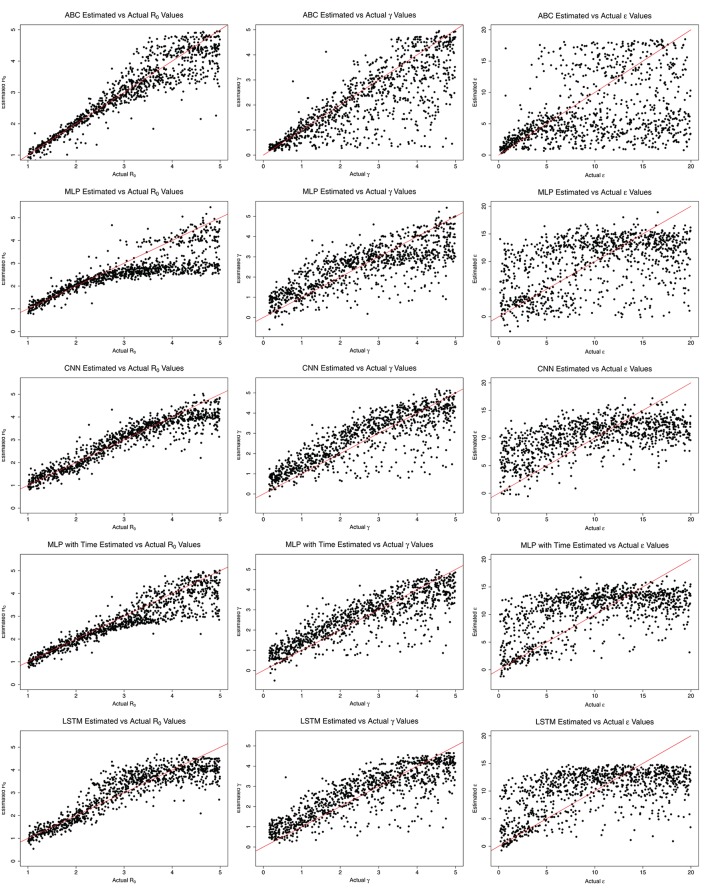
The estimated and actual parameter values from ABC, MLP, CNN, MLP with time, and LSTM.

Figure 4 shows the estimated parameter values compared to the actual values for each method. ABC estimates agree closely with the actual values for *R*_0_ < 3.0, with increasing error as *R*_0_ grows beyond 3.0. MLP also estimates *R*_0_ close to the actual values for *R*_0_ < 3.0. After *R*_0_ = 3.0, however, the estimate by MLP is nearly constant at a little <3.0. CNN estimates *R*_0_ close to the actual values while *R*_0_ is <4.0. MLP with time's trend is similar to ABC, with estimates near actual values for *R*_0_ < 3.0. LSTM shows close estimation for *R*_0_ < 2.0, then overestimates from 2.0 to 4.0, then underestimates for *R*_0_ > 4.0. Overall, the ML methods appear to underestimate *R*_0_ values, whereas ABC shows equal over- and underestimates. The ML methods show larger over- and underestimation than ABC for low *R*_0_.

All ML methods generally overestimate all values for γ. LSTM, MLP with time, and CNN estimates were close for all γ, while MLP again began estimating a constant, approximately 3.0 days^−1^ for γ > 3.0 days^−1^. ABC closely estimates γ values <2.0 days^−1^ and ϵ values <5.0 days^−1^. ABC, and to a lesser extent MLP with time and LSTM, appear to estimate ϵ somewhat closely for ϵ <5.0 days^−1^, but for values >5.0 days^−1^ the results for all methods are nearly random.

### Run times

The time to complete each method is shown in Table 1. The ABC method took 410 min to calculate the distances and accept/reject 1,000 test datasets from 100,000 training datasets. The time for a single dataset to be compared against 100,000 training datasets was 0.65 min. The fastest ML model, MLP, took 68 min to train while the slowest, LSTM, took 364 min. MLP with time and CNN took 71 and 184 min to train, respectively. This makes training of the ML models between 1.1 and 6.0 times faster than the time to verify a similar ABC method on 100,000 training datasets. Testing on a single dataset for the ML models took 0.03, 0.03, 0.05, and 0.06 min for MLP, MLP with time, CNN, and LSTM, respectively. These times are between 10.8 and 21.7 times faster than the estimate calculation for a single sample via ABC. When the size of the training set is increased from 100,000 to one million, the time required to estimate the parameters for a single ABC test set scales linearly with the number of comparisons, however the estimation via machine learning remains constant regardless of the number of samples used to train the data (Table 2).

**Table 2:** The time required to train one million samples and test on a single sample for the ABC, MLP, CNN, and LSTM methods.

**Method**	**Train (min)**	**Test (min)**
ABC	3,962	4.75
MLP	420	0.03
MLP with time	531	0.03
CNN	747	0.05
LSTM	784	0.06

### Application to real-world epidemiological data

We also compared ABC and ML with epidemiological data for mumps, measles and influenza.

*R*_0_ for mumps has been estimated between 3.6 and 4.5 (Edmunds et al., 2000). Table 3 shows the comparison of ML and ABC estimations of *R*_0_ with the typical real-world values. MLP, MLP with time, and CNN estimated *R*_0_ at approximately 4.0 using the data from Sullivan et al. (1985) for an outbreak in Centerville, OH, USA. ABC estimated *R*_0_ as slightly lower at 3.74, while LSTM greatly underestimated *R*_0_ at 2.71. These values were created based on an estimation of the effective reproductive number and the vaccine coverage of students within the school of 72.7%.

**Table 3:** The estimation results for *R*_0_ from previous published research on mumps and ABC, two MLP, CNN, and LSTM models.

**Methods**	***R*_0_ for mumps**
Previous study	3.6–4.5 (Edmunds et al., 2000)
ABC	3.74 (1.21, 12.09)
MLP	4.07 (1.10, 9.08)
MLP with time	3.92 (3.41, 4.25)
CNN	4.21 (2.53, 4.76)
LSTM	2.71 (1.83, 4.54)

*Estimates shown include the 95% credible/confidence intervals*.

For an outbreak of measles at Wincrange, Luxembourg (Mossong and Muller, 2000), the effective reproductive number was estimated as 1.5 (95% CI: 0.9, 2.2). Table 4 shows the comparison of ML and ABC estimations with the effective reproductive number estimated for the outbreak. All five of our methods underestimated the effective reproductive number of measles, but their estimates fell within the 95% CI estimated by the previous study.

**Table 4:** The estimation results for effective reproduction number from previous published research on measles and ABC, two MLP, CNN, and LSTM models.

**Methods**	**Effective reproduction number of measles**
Previous study	1.5 (Mossong and Muller, 2000)
ABC	1.16 (0.59, 4.88)
MLP	1.00 (0.63, 1.88)
MLP with time	0.91 (0.69, 1.07)
CNN	0.96 (0.19, 1.26)
LSTM	1.08 (0.80, 1.52)

*Estimates shown include the 95% credible/confidence intervals*.

The estimated *R*_0_ of an outbreak of influenza at a high school in New York during the 2009 influenza pandemic was 1.23 (Lessler et al., 2009). Table 5 shows the comparison of ML and ABC estimations of *R*_0_ with the effective reproductive number estimated for the outbreak. ABC estimated *R*_0_ nearly exactly at 1.24. MLP with time slightly underestimated the value at 1.06 (95% CI: 0.86, 1.30). Standard MLP greatly underestimated *R*_0_ at 0.40, while CNN and LSTM greatly overestimated the *R*_0_ at 2.84 and 1.84, respectively.

**Table 5:** The estimation results for *R*_0_ from previous published research on influenza and ABC, two MLP, CNN, and LSTM models.

**Methods**	***R*_0_ for influenza**
Previous study	1.23 (Lessler et al., 2009)
ABC	1.24 (0.96, 1.74)
MLP	0.40 (0.00, 0.79)
MLP with time	1.06 (0.86, 1.30)
CNN	2.84 (2.08, 3.35)
LSTM	1.84 (1.38, 3.54)

*Estimates shown include the 95% credible/confidence intervals*.

Based on the general analysis for various *R*_0_ values shown in Figures 1–3, most of the results above agree. For the mumps outbreak, the effective reproductive numbers were estimated around 1.0–1.2. At this low range of *R*_0_, based on Figure 1, the average error for all methods is relatively low, though LSTM and ABC have higher average errors than the other methods and also exhibit a small tendency to underestimate *R*_0_ for values near 1.0. All five methods also underestimated the effective reproduction number for measles, which was estimated at 1.5. With the exception of MLP with time, the methods contained 1.5 in their 95% CI intervals. However, the estimates for all five methods were within the 95% CI of the previous study. Finally, for the flu estimates, CNN and LSTM greatly overestimate *R*_0_ as 2.84 and 1.84, respectively. LSTM both over- and underestimates values around *R*_0_ = 1.2. The estimation of 2.84 by CNN is not robust with expected values, as though CNN does tend to overestimate more than underestimate at *R*_0_ = 1.2, an *R*_0_ of 2.84 is outside its average error window.

## Discussion

In this study we applied ML methods to estimate the epidemiological parameters of infectious diseases, and compared their accuracy and speed with ABC. In general, the width of confidence intervals estimated by ML are smaller than the credible intervals estimated by ABC. The average error of ML estimates are similar to ABC for *R*_0_, and larger for small values, but smaller for large values of γ and ϵ. Furthermore, the ML models were faster to train than ABC.

ABC was more robust to changes in the data, as shown in Tables 3–5. MLP with time was the most robust of the ML methods, with a tendency to underestimate *R*_0_. Given the difference in calculation times between ABC and MLP with time (410 min for ABC compared to 71 min for MLP to train 100,000 samples and 3,962 min for ABC compared to 531 min for MLP on one million samples), it is worth exploring methods which can reduce the underestimation of *R*_0_ in the MLP with time solution. Possibilities include increasing the amount of training data, hyperparameter tuning, increasing the depth of the model, and other general ML tuning methods which may be applied (Bishop, 2006). The ML methods estimated γ well, but with an obvious overestimation bias which can be observed in Figure 4, which may also be corrected by applying the previously mentioned approaches.

Interestingly, the point at which ABC is better than ML shifts with increasing sample size for γ and ϵ. When trained on one million datasets, this point decreased from <2.0 to 1.6 days^−1^ for γ and from <7.5 to 6.5 days^−1^ for ϵ. CNN and MLP with time showed similar estimation capability with ABC for *R*_0_, with average errors around 0.2 for *R*_0_ < 3.0 and increasing with increasing *R*_0_. The MLP and LSTM approaches showed poor estimation ability for *R*_0_.

While the ML models were faster to train than ABC was to verify, it should be noted that ABC verification of varying parameter values is not required, but ML training on all parameter values is necessary (Palmer and Chakravarty, 2014). That is, an ABC test estimate can be made in approximately <1 min without thorough verification of the general efficacy of the method, while the ML models must be fully trained before calculating a test estimate. However, once trained, the ML models do not need to be retrained unless there is a large amount of new data or some other reason arises to retrain the model (Bishop, 2006).

The problem of parameter estimation explored in this paper can be classified as a ML regression problem where the values of the estimates are continuous. The vast majority of ML research is on classification problems with discrete solutions and therefore “right” and “wrong” answers (Dreiseitl and Ohno-Machado, 2002). In bioinformatics and medicine, another characteristic of a large amount of ML solutions is the use of 2-dimensional images, again typically for classification, for example identifying breast cancer or analyzing MRIs (Sahiner et al., 1996; Chaplot et al., 2006; Krizhevsky et al., 2012). One example of ML being used for regression comes from the European Space Agency (ESA) (Verrelst et al., 2012; Caicedo et al., 2014), where neural networks and regression methods were explored for use in analyzing the large quantity of data being returned by the Sentinal-2 and Sentinel-3 satellites searching for life on far planets. Overall, the application of ML methods on regression problems requires further analysis to improve accuracy.

Note that the *R*_0_ value of 1.2 cited from the paper by Lessler et al. (2009) is the estimate made over the entire course of the outbreak. This value agrees with existing genetic analysis of the virus, as well as additional epidemiological studies which estimated the *R*_0_ value of influenza A(H1N1)pdm09 between 1.4 and 1.6 (Fraser et al., 2009).

Several disadvantages of ML for estimation of epidemiological parameters were found. First, ML approaches are highly sensitive to the size of parameter ranges (Ma et al., 1994). As parameter ranges increase, accuracy of the estimates decreases. In additional tests, we used normalization and standardization to try to reduce the impact of range size on model estimatibility, with limited success (Ioffe and Szegedy, 2015). Moreover, ML is not robust to changes in the initial condition of the model (Kolen and Pollack, 1991), even outside the parameters of interest, though ABC, too, showed sensitivity to initial conditions. This sensitivity may be reducible with larger datasets, deeper models, or the introduction of pruning algorithms and may be explored in later papers (Berthold and Hand, 2003).

In this paper, we have explored a single set of continuous time data, capturing parameters as constant values in a mathematical model. The transmission process of infectious disease does not strictly follow mathematical models and parameters can change values over time. Two approaches for future work which would partially address these issues are (1) a “discrete time analysis” to observe changes in parameter values over time and (2) testing the robustness of our estimates by checking values from different epidemiological models. “Discrete time analysis” is a discretization of the time component of our model with the assumption that the parameter values are constant between time intervals to observe changes in parameter values over time. To check the robustness of our estimates, but still using simulated data, we could create multiple datasets from the Susceptible-Exposed-Infectious-Removed-Susceptible (SEIRS) (Cooke and van den Driessche, 1996) epidemiological model and use this much more complex data to test a model trained from simpler SIR or SEIR model data. This would check the robustness of the systems to changes in unknown parameters and allow us to observe and estimate the sensitivity of our systems. Furthermore, using an SEIRS data model for data generation would allow for analysis of longer and recurring epidemics (Cooke and van den Driessche, 1996) and the efficacy of ML and ABC in estimating more complex disease dynamics.

In conclusion, we have confirmed that both ABC and ML can estimate SEIR model parameters, with ABC and MLP with time being the most robust methods for different SEIR models and parameters. ML models learn more quickly than ABC can be verified, however ABC verification is highly parallelizable, i.e., the problem can be broken into several processes and estimated concurrently, while the learning time for ML models is more difficult to reduce. A key benefit of ML is the speed with which new datasets can be analyzed. A single, new sample can be analyzed in a few seconds, compared to several minutes by ABC, and is constant regardless of the number of datasets used for training. This means a trained ML model would be helpful when estimating large batches of new data.

## Author contributions

RO and HT conceived the experiments; HT, RO, and KI participated in discussions; HT and RO conducted the experiments; RO and HT analyzed the results; HT, RO, and KI wrote the manuscript. All authors reviewed the manuscript.

### Conflict of interest statement

The authors declare that the research was conducted in the absence of any commercial or financial relationships that could be construed as a potential conflict of interest.

## References

[B1] Bergstra J. Breuleux O. Bastien F. Lamblin P. Pascanu R. Desjardins G. (2010). Theano: A cpu and gpu math compiler in python, in Proceedings of the 9th Python in Science Conference, (Austin, TX), 1–7.

[B2] Berthold M. Hand D. J. (2003). Intelligent Data Analysis: An Introduction. Verlag: Springer Science & Business Media.

[B3] Bishop C. M. (2006). Pattern Recognition and Machine Learning. New York, NY: Springer.

[B4] Bjrnstad O. N. Finkenstdt B. F. Grenfell B. T. (2002). Dynamics of measles epidemics: estimating scaling of transmission rates using a time series sir model. Ecol. Monogr. 72, 169–184. 10.1890/0012-9615(2002)072[0169:DOMEES]2.0.CO;2

[B5] Caicedo J. P. R. Verrelst J. Muoz-Mar J. Moreno J. Camps-Valls G. (2014). Toward a semiautomatic machine learning retrieval of biophysical parameters. IEEE J. Sel. Top. Appl. Earth Obs. Remote Sens. 7, 1249–1259. 10.1109/JSTARS.2014.2298752

[B6] Chaplot S. Patnaik L. Jagannathan N. (2006). Classification of magnetic resonance brain images using wavelets as input to support vector machine and neural network. Biomed. Signal Proc. Control 1, 86–92. 10.1016/j.bspc.2006.05.002

[B7] Cooke K. L. van den Driessche P. (1996). Analysis of an seirs epidemic model with two delays. J. Math. Biol. 35, 240–260. 10.1007/s002850050051 9008370

[B8] Diekmann O. Heesterbeek J. Roberts M. (2009). The construction of next-generation matrices for compartmental epidemic models. J. R. Soc. Interface 7, 873–885. 10.1098/rsif.2009.0386 19892718 PMC2871801

[B9] Diekmann O. Heesterbeek J. A. P. Metz J. A. (1990). On the definition and the computation of the basic reproduction ratio r0 in models for infectious diseases in heterogeneous populations. J. Math. Biol. 28, 365–382. 10.1007/BF00178324 2117040

[B10] Dreiseitl S. Ohno-Machado L. (2002). Logistic regression and artificial neural network classification models: a methodology review. J. Biomed. Inform. 35, 352–359. 10.1016/S1532-0464(03)00034-0 12968784

[B11] Edmunds W. J. Gay N. J. Kretzschmar M. Pebody R. G. Wachmann H. (2000). The pre-vaccination epidemiology of measles, mumps and rubella in europe: implications for modelling studies. Epidemiol. Infect. 125, 635–650. 10.1017/S0950268800004672 11218214 PMC2869647

[B12] Fraser C. Donnelly C. A. Cauchemez S. Hanage W. P. Van Kerkhove M. D. Hollingsworth T. D.. (2009). Pandemic potential of a strain of influenza a (h1n1): early findings. Science 324, 1557–1561. 10.1126/science.1176062 19433588 PMC3735127

[B13] Hochreiter S. Schmidhuber J. (1997). Long short-term memory. Neural Comput. 9, 1735–1780. 10.1162/neco.1997.9.8.1735 9377276

[B14] Ioffe S. Szegedy C. (2015). Batch normalization: Accelerating deep network training by reducing internal covariate shift, in Proceedings of the 32nd International Conference on Machine Learning, Vol. 37, eds Bach F. Blei D. *Proceedings of Machine Learning Research*, (Lille: PMLR), 448–456.

[B15] Kolen J. F. Pollack J. B. (1991). Back propagation is sensitive to initial conditions, in Advances in Neural Information Processing Systems (Cambridge, MA), 860–867.

[B16] Krizhevsky A. Sutskever I. Hinton G. E. (2012). Imagenet classification with deep convolutional neural networks, in Advances in Neural Information Processing Systems 25, eds Pereira F. Burges C. J. C. Bottou L. Weinberger K. Q. (Cambridge, MA: Curran Associates, Inc.), 1097–1105.

[B17] Lessler J. Reich N. G. Cummings D. A. the New York City Department of Health, and Team, M. H. S. I. I. (2009). Outbreak of 2009 pandemic influenza a (h1n1) at a new york city school. N. Engl. J. Med. 361, 2628–2636. 10.1056/NEJMoa0906089 20042754

[B18] Ma H. El-Keib A. A. Ma X. (1994). Training data sensitivity problem of artificial neural network-based power system load forecasting, in Proceedings of 26th Southeastern Symposium on System Theory (New York, NY), 650–652.

[B19] Maas A. L. Hannun A. Y. Ng A. Y. (2013). Rectifier nonlinearities improve neural network acoustic models, in Proceedings of 30th International Conference on Machine Learning, Vol. 30 New York, NY.

[B20] Magal P. Ruan S. (2014). Susceptible-infectious-recovered models revisited: from the individual level to the population level. Math. Biosci. 250, 26–40. 10.1016/j.mbs.2014.02.001 24530806

[B21] Mossong J. Muller C. (2000). Estimation of the basic reproduction number of measles during an outbreak in a partially vaccinated population. Epidemiol. Infect. 124, 273–278. 10.1017/S0950268899003672 10813153 PMC2810911

[B22] Nishiura H. Castillo-Chavez C. Safan M. Chowell G. (2009). Transmission potential of the new influenza a (h1n1) virus and its age-specificity in japan. Euro Surveill 14:19227. 10.2807/ese.14.22.19227-en 19497256

[B23] Palmer J. Chakravarty A. (2014). Supervised machine learning, in An Introduction To High Content Screening: Imaging Technology, Assay Development, and Data Analysis in Biology and Drug Discovery, eds Haney S. A. Bowman D. Chakravarty A. (Hoboken, NJ: John Wiley & Sons, Inc.), 231.

[B24] Sahiner B. Chan H.-P. Petrick N. Wei D. Helvie M. A. Adler D. D.. (1996). Classification of mass and normal breast tissue: a convolution neural network classifier with spatial domain and texture images. IEEE Trans. Med. Imaging 15, 598–610. 10.1109/42.538937 18215941

[B25] Saulnier E. Gascuel O. Alizon S. (2017). Inferring epidemiological parameters from phylogenies using regression-abc: a comparative study. PLOS Comput. Biol. 13:e1005416. 10.1371/journal.pcbi.1005416 28263987 PMC5358897

[B26] Sokolova M. Japkowicz N. Szpakowicz S. (2006). Beyond Accuracy, F-Score and ROC: A Family of Discriminant Measures for Performance Evaluation. Berlin; Heidelberg: Springer Berlin Heidelberg, 1015–1021.

[B27] Sullivan K. M. Halpin T. J. Marks J. S. Kim-Farley R. (1985). Effectiveness of mumps vaccine in a school outbreak. Am. J. Dis. Child. 139, 909–912. 10.1001/archpedi.1985.02140110063030 4036925

[B28] Sunnåker M. Busetto A. G. Numminen E. Corander J. Foll M. Dessimoz C. (2013). Approximate bayesian computation. PLOS Comput. Biol. 9:e1002803. 10.1371/journal.pcbi.1002803 23341757 PMC3547661

[B29] Verrelst J. Muñoz J. Alonso L. Delegido J. Rivera J. P. Camps-Valls G. (2012). Machine learning regression algorithms for biophysical parameter retrieval: Opportunities for sentinel-2 and -3. Remote Sens. Environ. 118, 127–139. 10.1016/j.rse.2011.11.002

[B30] Vynnycky E. Trindall A. Mangtani P. (2007). Estimates of the reproduction numbers of spanish influenza using morbidity data. Int. J. Epidemiol. 36, 881–889. 10.1093/ije/dym071 17517812

